# Obesity as a Risk Factor for Severe COVID-19: Summary of the Best Evidence and Implications for Health Care

**DOI:** 10.1007/s13679-021-00448-8

**Published:** 2021-08-10

**Authors:** Naveed Sattar, Jonathan Valabhji

**Affiliations:** 1 Institute of Cardiovascular and Medical Sciences, BHF Glasgow Cardiovascular Research Centre, University of Glasgow, 126 University Place, Glasgow, G12 8TA UK; 2 NHS England & Improvement, London, UK; 3 Department of Diabetes and Endocrinology, St Mary’s Hospital, Imperial College Healthcare NHS Trust, London, UK; 4 Division of Metabolism, Digestion and Reproduction, Imperial College London, London, UK

**Keywords:** Epidemiology, Body mass Index, Genetics, Multimorbidity, Diet, Activity

## Abstract

**Purpose of Review:**

To collate the best evidence from several strands—epidemiological, genetic, comparison with historical data and mechanistic information—and ask whether obesity is an important causal and potentially modifiable risk factor for severe COVID-19 outcomes.

**Recent Findings:**

Several hundred studies provide powerful evidence that body mass index (BMI) is a strong linear risk factor for severe COVID-19 outcomes, with recent studies suggesting ~5-10% higher risk for COVID-19 hospitalisation per every kg/m^2^ higher BMI. Genetic data concur with hazard ratios increasing by 14% per every kg/m^2^ higher BMI. BMI to COVID-19 links differ markedly from prior BMI-infection associations and are further supported as likely causal by multiple biologically plausible pathways.

**Summary:**

Excess adiposity appears to be an important, modifiable risk factor for adverse COVID-19 outcomes across all ethnicities. The pandemic is also worsening obesity levels. It is imperative that medical systems worldwide meet this challenge by upscaling investments in obesity prevention and treatments.

## Evidence for Obesity as a Risk Factor for Severe COVID-19

This paper first discusses the range of evidence implicating obesity as a causal risk factor for adverse COVID-19-related outcomes (summarised Table 1). It then discusses why such evidence, when added to widening appreciation of the adverse impacts of obesity on many chronic conditions, as well as the impacts of lockdown on body mass index (BMI) levels, should stimulate relevant health systems to upscale efforts on obesity prevention and management.

**Table 1:** A summary of the range of evidence linking obesity to adverse COVID-19 outcomes following SARS-CoV2 infection

	Current evidence	Future research ideas
Epidemiological	Multiple studies—of varying veracity—show higher BMI broadly linearly linked to worse COVID-19 outcomes, including at least a dozen meta-analyses. Where examined, links between BMI and mortality are stronger than links between BMI and non-COVID-related mortality, as well as far stronger than prior links between BMI and influenza or adult respiratory distress syndrome (ARDS).	More evidence of links of differing adiposity measures to COVID-19 would be useful.
Genetic	A recent Mendelian randomisation study in those of European Ancestry found obesity to be a likely causal risk factor with a 14% (95% CI 7 to 21%) higher risk for hospitalisation due to COVID-19 per every 1kg/m^2^ higher BMI. No other causal risk factors were identified though more power needed in future studies.	Validation of these genetics’ findings will come soon and should expand to other measures of adiposity and in differing ethnic groups.
Interventions	There are no trials as such, but a retrospective bariatric surgery analysis suggests people who lose (around 12 BMI units) weight through bariatric surgery are at 69% lower risk of hospitalisation and lower risk for ICU admission than pre-surgery BMI-comparable people who had not undergone surgery.	It remains unclear whether formal trials of weight loss can be large enough to prove intentional weight loss protects against adverse COVID-19 outcomes. However, it may be possible that randomised trials of weight loss-promoting drugs, if enough people in trials did develop COVID-19 and robust outcome data were recorded, could prove the weight loss theory.
Biological plausibility	Obesity contributes to metabolic derangements, worse lung and kidney function, poorer vascular health, and is associated with a low-grade inflammation and a greater thrombogenic potential. In this way, obesity lessens an individual’s capacity to cope with the systemic effects of the hyperimmune response.	Understanding how excess fat stores or adipose tissue impairs the immune response, in general and in COVID-19, requires dedicated research

## Epidemiology

There is now overwhelming evidence that BMI relates linearly to adverse COVID-19 outcomes. A quick literature search on BMI and COVID comes up with over 500 papers, and though many are of suboptimal quality, there is a consistent finding of higher BMI being associated with worse COVID-19 outcomes. Some of the best data comes from larger nationwide cohort studies. For example, the OpenSAFELY study of over 17 million adults in England, showed that a body mass index (BMI) of over 40 to be associated with a near doubling [HR 1.92 (1.72–2.13)] of death from COVID-19 compared to people who were not living with obesity in a model adjusted for confounders [1]. In UK biobank studies, whilst not nationally representative, we showed that BMI, measured around a decade before the pandemic began, which has its advantages as BMI measured when younger is more likely to be accurate and relate to adverse outcomes [2], was linearly associated with higher risk for hospitalisation and death from COVID-19 [3•]. Moreover, associations between BMI and adverse COVID-19 outcomes appeared stronger in younger people and those of non-white ethnicity [3•]. This latter finding was recently extended by Jebb and colleagues in a recent analysis of QResearch data with almost seven million people [4••]. The authors demonstrated a linear increase in risk beyond a BMI of 23 kg/m^2^ with hospitalisation risk increasing by an HR (adjusted) of 1.05 [95% CI 1.05–1.05]) per kg/m^2^. Once again, associations were stronger in younger people, but in this case, risks were higher in those of Black ethnicity. In South Asians, the data also showed higher risks for death and hospitalisation per unit increase in BMI but, perhaps due to lack of power, not significantly so. Indeed, the study was limited by BMI availability in routine health care, which is by no means comprehensive, resulting in lower power in some ethnic groups.

The wealth of evidence is such that more than a dozen meta-analyses have been conducted on this topic, for example [5, 6]. The reported levels of risk, however, vary somewhat between studies as differing study populations—population based versus hospital based—can lead to differing findings with the latter being less informative of risks in the general population as patients in hospital with COVID represent a high-risk group with more severe manifestations, and decisions to admit to hospital are influenced by varying, often health system specific, factors. In addition, differing choices of the referent BMI category, varying adjustment models and outcomes, plus other discrepancies in methods, means it is not always possible to compare BMI-to-COVID risk levels between studies. Even so, the totality of evidence supports higher BMI being associated with a greater chance of SARS-CoV2 infection, greater likelihood of hospital admission and greater need for mechanical ventilation and mortality from COVID-19. The evidence, where available in specific subgroups such as those with diabetes [7], suggests BMI may be more strongly linked to COVID-19 mortality than mortality from other causes, an important observation which further highlights the strength of the link between obesity and COVID-19. As such, if elevated BMI is causally related to adverse outcomes, it represents a key modifiable *pre-infection* risk factor to lessen chances of developing severe COVID-19.

## Genetics

Given the potential for confounding in observational studies and the problem with BMI in the setting of multimorbidity—BMI is often lowered in many conditions due to reverse causality—additional evidence is needed to support BMI as causal for adverse COVID-19 outcomes. Mendelian randomisation (MR) is one such method. It exploits genes randomly allocated at conception which leads people to have lifelong lower or higher BMI levels. By examining these genes and relating them to long term outcomes, a more informed causal case between excess weight and disease outcomes can be made. Such methods have been used to support higher BMI as a causal risk factor for multiple cardiovascular outcomes (e.g., coronary heart disease, atrial fibrillation and heart failure) [8•], and many other diseases. Using this MR approach, Leong and colleagues related genetics that underpin 17 cardiometabolic traits to risk for COVID-19 outcomes [9••]. The only statistically positive association was for genes that predict higher BMI—a 14% (95% CI 7 to 21%) higher risk for COVID-19 hospitalisation per every 1kg/m^2^ higher BMI level was noted, a risk around twice seen in observational studies. Such risk seemed to be explained in part by the downstream links of BMI to several conditions including type 2 diabetes, chronic kidney disease, coronary artery disease and stroke, which makes sense as obesity is a causal risk factor for such conditions. These MR data, conducted in those with European ancestry, therefore add strong support for elevated BMI being causally related to COVID-19 severity, and perhaps at a magnitude that is a little stronger than suggested by observational data. However, further genetic data are needed to validate findings and, to extend analyses to other ethnicities and to examine whether other measures of adiposity have similar or potentially stronger links to COVID-19 mortality.

## Interventions

Given that the pandemic began just over a year ago, it would be unrealistic to expect adequately powered randomised trials of weight loss to have been conducted to prove weight loss leads to lower risks for severe COVID-19. However, indirect findings from patients before and after bariatric surgery are notable. Aminian and colleagues identified 33 patients in their records with prior bariatric surgery who had tested positive for SARS-CoV-2 and matched them to 330 people who had a BMI ≥40 kg/m^2^ at the time of a positive viral test but who had not undergone surgery [10]. The surgery patients had a BMI near 49 kg/m^2^ before surgery which went down to around 37 kg/m^2^ at the time of SARS-CoV-2 testing, whereas the controls had a BMI of around 46 kg/m^2^. The authors reported the surgery group to have around a 69% (95% CI 12-89%) lower risk for admission to hospital compared to the controls. Also, none in the surgery group required ICU admission, whereas 13% of the control population did. Admittedly, this observational analysis had the usual limitations of such research but, even so, it does suggest intentional weight loss may lessen risks of severe COVID-19 in those people living with severe obesity, an important observation since, as described below, social factors may also put heavier people at risk of COVID-19 infection. Whether smaller amounts of intentional weight loss benefit others across the adiposity spectrum remains to be proven.

## Mechanisms

The potential mechanisms that link obesity to higher risk for COVID-19 severity are likely to be multiple-fold, as we recently reviewed [11••]. First, given the known association between obesity and socioeconomic status, there is a greater risk for people living with obesity being infected in the context of multiple-person households; and people who are heavier exhale substantially more virus as they breathe out [12•]. Secondly, once infected, the chances of experiencing the second phase hyperimmune response, responsible for severe disease, may also depend on carrying relative more fat tissue as there may be a critical interplay between adipose tissue and immunity as fat cells can liberate multiple inflammatory proteins. This latter suggestion is speculative, however, and requires further research. Finally, as obesity contributes to metabolic derangements (linked in turn to ectopic fat in the liver and pancreas), poorer vascular health, and greater thrombotic potential, and can also adversely impact lung and renal function, people living with obesity have far less buffering capacity against the systemic effects of the hyperimmune response [11••]. There may also be indirect links between obesity and COVID-19 outcomes such as an adverse diet-induced microbiome impact on immune efficiency.

## Evidence Comparing Risk Patterns for COVID-19 Mortality to Those from Historical Observations

It is notable that the link between type 2 diabetes and mortality risk from COVID-19 is particularly strong, as reported in several studies. Where examined, this diabetes to COVID-19 mortality link is also meaningfully stronger than comparable risks in people with existing cardiovascular conditions [13], which is notable as one would normally have anticipated life years lost from diabetes to be like people with existing cardiovascular conditions [14]. As obesity is a much stronger upstream risk factor for type 2 diabetes than for cardiovascular conditions, and as type 2 diabetes can be prevented or remission achieved by rapid weight changes, weight changes could lessen COVID-19 risks by preventing some from converting to diabetes. Even if glycaemia levels do not change, intentional weight loss is well known to favourably impact blood pressure and lipid levels as well as cardiorespiratory fitness, plus partially reverse many of the other pathways discussed above. These findings suggests that any lifestyle improvements to lessen weight intentionally could protect people from a more severe outcome should they contract SARS-CoV-2.

At the same time, and as also nicely summarised by Jebb and colleagues in their recent paper [4••], prior work on seasonal influenza infection in the USA showed that obesity (BMI ≥30 kg/m^2^) is not a risk factor for requiring mechanical ventilation or death [15]. Furthermore, there is no clear evidence from a meta-analysis of over 6000 patients that obesity was associated with higher mortality in patients admitted to the ICU with adult respiratory distress syndrome [16]. These findings suggest that excess weight has a specific association with risk in people admitted with COVID-19, which may be a function of the hyperimmune response that stresses the body in a manner quite distinct to influenza or related conditions.

## Directions for Future Research

Given the wealth of evidence linking BMI to adverse COVID-19 outcomes, further epidemiological research would usefully examine adiposity-COVID links across differing ethnicities (Table 1). Further work examining whether measures of central adiposity (i.e., waist or waist to hip ratio) give better estimates of risks of excess (regional) adiposity from COVID-19, would also be useful. Additional genetic data to validate early findings from Leong and colleagues [9••] would also be valuable, including extending results to differing ethnic groups and to genetic variants linked to measures of body fat distribution. With respect to interventions, it is unlikely that a weight loss trial can be adequately powered or sufficiently large enough to prove benefits of intentional weight loss on COVID-19 risks. It is also unlikely that ongoing trials of novel weight losing drugs have sufficient power to show benefits of drug-induced weight loss on COVID-19 outcomes but, even so, such data would be of interest. Finally, as this COVID-19 pandemic has shone a light on potential links between excess fat and immune function, more research examining such links at a molecular level would be valuable (Table 1).

## The Urgent Case for Better Obesity Prevention and Management

### Why Now?

**Fig. 1:**
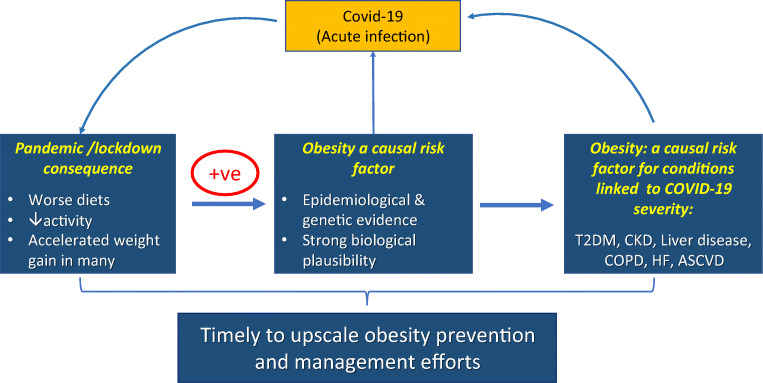
Obesity and COVID-19. Evidence from multiple sources now links excess adiposity to risk for severe COVID-19 outcomes. Additionally, many conditions associated with adverse COVID-19 outcomes (e.g. type 2 diabetes, COPD) are also strongly linked to excess adiposity. At the same time, the pandemic and related lockdowns and changes in life circumstances are accelerating weight gain in many in the population. All of these facts means that governments around the world, and particularly in countries where obesity levels are already high, need to prioritise obesity prevention and management efforts. The consequences of not doing so will be high for both people and economies

If ever there was a time to discuss obesity management more widely, it is now. The COVID-19 pandemic has shone a light on obesity in an apparent new way—i.e., as a risk factor for an *acute* infectious condition that has killed more than 3 million people worldwide as well as contributing to many experiencing long COVID. Whilst we know that people who are living with overweight or obesity tend to do worse with some infections, the new COVID-19-obesity data, allied to an increasing appreciation of the role of obesity in many *chronic* conditions, presents a real opportunity to bolster the prevention and management of obesity (Figure1). It should also be noted that obesity is also a major risk factor for multimorbidity. Survival rates from many obesity-related conditions have improved, due to better medicines and interventions that lessen mortality risks but do not necessarily lessen weights or lessen associated morbidity. This means more people are living longer with their obesity and so can develop multiple obesity-related conditions as they age.

## Pandemic Has Likely Worsened Obesity Levels

The indirect consequences of the pandemic have paradoxically, made the situation even worse as more people have worsened than improved their diets [17], and less people are as active as they were pre-pandemic, linked to more working from home, so less active travel, as well as anxieties in many individuals in spending time outdoors. There is emerging evidence to suggest pandemic-associated weight gain, potentially more marked in younger people, in women compared to men, and in people from more deprived communities [18•, 19].

## Roles for National Governments, Local Governments, and Health Care Systems

So, what can be done? Debate has often focussed on the relative merits of population-level vs. individual-level interventions, the former associated with large reach and very small cost and impact per individual, and the latter associated with more limited reach and higher cost and impact per individual. We would argue that both approaches have merit and should be embraced—any single approach is likely to only carry a small effect size, and so we need as many different policies as possible in our portfolio of actions to make an appreciable difference. Population-level interventions often fall within the remit of national and local governments, with respective examples: levies on sugar sweetened beverages [20]; and local controls over establishing unhealthy food outlets near schools [21]. Individual-level interventions are often embedded in health care systems, involving facilitated access for individuals to interventions supporting the achievement of a healthy weight [22].

Recent announcements by the UK Government are consistent with this broad portfolio of actions, involving both population-level and individual-level interventions [23, 24]. This includes proposals: to ban television and online adverts for food high in fat, sugar and salt before 9pm; to end deals like ‘buy one get one free’ on unhealthy food high in salt, sugar and fat; to cause calories to be displayed on menus to help people make healthier choices when eating out; to ensure that alcoholic drinks have to list hidden ‘liquid calories’; to provide significant additional investment to support individuals—children, adults and families—achieve and maintain a healthier weight. Ongoing evaluation of the impact of such policies would be important to determine whether more needs be done.

At the clinical level, more can be done to tackle obesity, which is upstream of many conditions. However, whilst most clinicians are adept at tackling its downstream consequences, few have the expertise to help their patients change their weight trajectories, and most rarely try, as they believe any such advice will inevitably fail. Yet, the evidence-based methods to help weight loss have improved over the last decade. For example, based on randomised trials [25], we know now that referral to commercial weight loss programmes can help many achieve meaningful weight loss, although uptakes need to improve, particularly in men who are usually at higher obesity-associated risks for many conditions, including COVID-19. However, there is more evidence to suggest that weight loss interventions implemented through national policy, with referral pathways involving General Practice, have the potential to overcome such inequities of access, across the dimensions of gender, ethnicity, and socioeconomic status [22, 26]. We also know that low calorie diets can help some shed many kilograms rapidly with the potential to achieve remission of diseases, such as type 2 diabetes [27].

There are now also better medications to help achieve meaningful weight loss (~10kg or more [28, 29]) and, though expensive, we may be at the start of an era where substantial weight loss is achievable by pharmacotherapy, in a manner that appears safe and hopefully lessens risks for obesity-related outcomes. Evidence from trials completing in the next few years, e.g. SURPASS [30] and SELECT [31], is eagerly anticipated. Also, bariatric surgery has its place for some patients living with obesity and comorbidities as it can transform and extend lives, and substantially reduce the risk of future complications, including cardiovascular disease, cancers, kidney disease and heart failure [32, 33].

## Gaps in Practice?

Despite all the above interventions, many people living with obesity or obesity-related comorbidities are falling through the net and, presently, only a minority are supported to achieve a healthier weight. Many are also not at the stage where they are willing to consider referral to outside agencies, and even though newer drugs for weight loss are coming, they are expensive, at different stages of licensing, and when they are stopped, most people put weight back on. Hence, we need to think outside the box and extent our current armamentarium to tackle obesity.

The National Health Service (NHS) in England has recently published intentions and started implementation to deliver new or expanded services: to provide a targeted support offer and access to weight management services for people with a diagnosis of diabetes or hypertension and living with a BMI in the obese range (adjusted appropriately for ethnicity); doubling of the capacity of the NHS Diabetes Prevention Programme to support participation of 200,000 individuals per year by 2024 [22], including digital options to widen patient choice and target inequality; test an NHS programme supporting low calorie diets for people living with obesity and type 2 diabetes, to try to achieve remission of type 2 diabetes through weight loss; and to expand nutritional education for health care professionals and trainees [34]. Many other countries are also considering or adopting diabetes prevention programmes.

In a recent paper, we described how in recent years there has been an increase in understanding of how to support people make sustainable lifestyle changes, particularly dietary [35•]. We further argued that such knowledge could be collated into a leaflet that could be easily explained and given to patients in many clinical settings (primary or secondary care) so that more health care professionals can give specific, actionable, evidenced-based advice that is consistent and easily digestible. We proposed that this simple leaflet, an extension of the ‘top ten tips’ concept [36], could detail a range of options, from simple dietary changes to more-intensive options. We were also careful to stress that such advice should always be given in an empathetic manner, as language really does matter when health care professions engage people living with obesity [37]. Of course, such work would need to be carefully audited or researched and could be improved over time.

There are also excellent online resources for weight management that can be accessed, such as that available via the NHS website [38, 39].

Finally, in a separate paper, we stressed that for many dietary changes to be sustained, patients should be encouraged to retrain their palates towards healthier, equally enjoyable, foods [40]. In other words, people must be told that positive dietary changes can take a little time to embed, and that perseverance is needed, but that the rewards are worthwhile. Not making such points represents a missed opportunity.

## Activity also Matters

Simpler messages should be usefully extended to activity tips. Here, people need to be told it is rarely possible to achieve weight loss by activity alone and that dietary improvements are often pivotal and should be started first or in conjunction. That said, activity helps lessen weight regain and most people can be encouraged to be more active, but once again, the messaging needs to be clearer and simple. For example, one piece of advice would be to walk more as 10 mins extra walking can achieve 1000 extra steps per day, helpful to many who are currently sedentary. Of course, for activity to benefit long-term, it needs to be sustained over time, and so the main concept is for people to find something they enjoy and try to form new habits along these lines. As the lockdown eases, it is hoped that activity levels will climb to slow population-wide weight gain.

## Conclusion

It is overwhelmingly clear that obesity (or excess ectopic fat) is an important and major risk factor for adverse COVID-19 outcomes across many ethnicities. Future evidence is unlikely to change this stark conclusion. Consequently, obesity becomes a strong ‘upstream’ risk factor not just for many *chronic* diseases but also for an *acute* viral pandemic that, sadly, continues to impact across the world, leaving considerable morbidity and mortality in its wake. Furthermore, in many places, the indirect consequence of the pandemic (worsening diets and less activity) will be increasing obesity prevalence, perhaps most manifest in those most at risk. Such changes will fuel even more obesity-related diseases, further stretching health services internationally. It is therefore important that health systems around the world meet this challenge by upscaling emphasis on and investments in obesity prevention and treatments.

Recent successful interventions in weight management need to be extended and offered to many more people at risk. In addition, new, simpler, innovative ways to spread evidence-based dietary or activity messages should also be examined as many people with risk factors are not currently receiving advice. If ever there was a time to improve obesity prevention and management, it is now.
